# Quantitative High-Throughput Screening Identifies 8-Hydroxyquinolines as Cell-Active Histone Demethylase Inhibitors

**DOI:** 10.1371/journal.pone.0015535

**Published:** 2010-11-23

**Authors:** Oliver N. F. King, Xuan Shirley Li, Masaaki Sakurai, Akane Kawamura, Nathan R. Rose, Stanley S. Ng, Amy M. Quinn, Ganesha Rai, Bryan T. Mott, Paul Beswick, Robert J. Klose, Udo Oppermann, Ajit Jadhav, Tom D. Heightman, David J. Maloney, Christopher J. Schofield, Anton Simeonov

**Affiliations:** 1 Structural Genomics Consortium, University of Oxford, Headington, United Kingdom; 2 Department of Biochemistry, University of Oxford, Oxford, United Kingdom; 3 National Institutes of Health Chemical Genomics Center, National Human Genome Research Institute, National Institutes of Health, Bethesda, Maryland, United States of America; 4 Department of Chemistry and the Oxford Centre for Integrative Systems Biology, University of Oxford, Oxford, United Kingdom; St. George's University of London, United Kingdom

## Abstract

**Background:**

Small molecule modulators of epigenetic processes are currently sought as basic probes for biochemical mechanisms, and as starting points for development of therapeutic agents. N^ε^-Methylation of lysine residues on histone tails is one of a number of post-translational modifications that together enable transcriptional regulation. Histone lysine demethylases antagonize the action of histone methyltransferases in a site- and methylation state-specific manner. N^ε^-Methyllysine demethylases that use 2-oxoglutarate as co-factor are associated with diverse human diseases, including cancer, inflammation and X-linked mental retardation; they are proposed as targets for the therapeutic modulation of transcription. There are few reports on the identification of templates that are amenable to development as potent inhibitors *in vivo* and large diverse collections have yet to be exploited for the discovery of demethylase inhibitors.

**Principal Findings:**

High-throughput screening of a ∼236,000-member collection of diverse molecules arrayed as dilution series was used to identify inhibitors of the JMJD2 (KDM4) family of 2-oxoglutarate-dependent histone demethylases. Initial screening hits were prioritized by a combination of cheminformatics, counterscreening using a coupled assay enzyme, and orthogonal confirmatory detection of inhibition by mass spectrometric assays. Follow-up studies were carried out on one of the series identified, 8-hydroxyquinolines, which were shown by crystallographic analyses to inhibit by binding to the active site Fe(II) and to modulate demethylation at the H3K9 locus in a cell-based assay.

**Conclusions:**

These studies demonstrate that diverse compound screening can yield novel inhibitors of 2OG dependent histone demethylases and provide starting points for the development of potent and selective agents to interrogate epigenetic regulation.

## Introduction

N^ε^-Methylation of lysine residues on histone tails is an important post-translational modification in transcriptional regulation. Lysine residues in histones are methylated and demethylated by sequence-specific methyltransferases and demethylases. The dynamic interplay between these, and other, enzyme classes is an important process in the control of chromatin structure and transcriptional activity (for review see [1], [2], [3]). Specific histone lysine methylation sites (e.g. histone H3K4 methylation) are, in general, associated with the promoters of actively transcribed genes [4], whereas other methylation sites (e.g. histone H3K9 methylation) are associated with heterochromatic regions of the genome [5]. A molecular understanding of the enzymes that place and remove histone modifications, and the proteins that bind to them, is only beginning to emerge. In several instances, mutations of genes encoding histone modifying enzymes have been linked with diseases, including cancers, mental retardation, and midline defects (*reviewed in* [6], [7], [8]).

Small-molecule inhibitors of the catalytic activity of histone-modifying enzymes are of interest both as therapeutic agents, as shown by the clinically-approved histone deacetylase inhibitors Vorinostat and Romidepsin [9], and as chemical probes for investigating biological function. Biological techniques such as RNA interference are useful in functional assignment work, but are of limited use when proteins have separate functional domains. Many histone-modifying enzymes contain various domains in addition to a catalytic domain, including those involved in DNA-binding and protein-protein interactions. There is thus an unmet need for the development of small molecule inhibitors of catalytic activity that allow the other functions of histone-modifying proteins to remain intact.

The jumonji-C (JmjC) domain-containing enzymes constitute the largest class of histone demethylases, and the family is predicted to include about 30 human members. These enzymes utilize Fe(II) in a 2-oxoglutarate-dependent dioxygenase mechanism to remove methyl groups from methylated lysines of histone tails. JmjC-domain demethylases are linked with diseases, including androgen-dependent prostate cancer [10], obesity [11], and X-linked metal retardation [12], suggesting that these enzymes may constitute novel targets for therapeutic intervention.

To date only few inhibitor scaffolds for the 2-oxoglutarate (2OG) dependent histone lysine demethylases have been described and the available structure-activity data are limited [13], [14], [15]. Identified inhibitors predominantly include 2OG mimetics, such as 2,4-pyridinedicarboxylic acid (PDCA), a fragment-size JmjC-domain inhibitor of micromolar potency, which needs to be modified to a prodrug form in order to enable penetration of cell membranes [16] and functionalised hydroxamic acids [17]. A recent screen of a small-size bioactives collection [18] yielded primarily flavonoid and catechol type molecules; these are known iron chelators and represent promiscuous inhibitors affecting a wide range of molecular targets. However, this work suggests that screening a large and diverse collection for chemotypes amenable to further optimization may be useful. Here we report the application of quantitative high-throughput screening (qHTS) [19] to identify new series of inhibitors of histone demethylases. A diverse collection of ∼236,000 compounds was screened in concentration-response format by using a real-time fluorogenic coupled assay designed to monitor formaldehyde production from the demethylation reaction. One of the hit series, the 8-hydroxyquinolines (8HQs), demonstrates clear structure activity relationships and is here presented as a good template from which selective inhibitors may be designed. Furthermore, we show that 8HQs inhibit JMJD2 via binding to the active-site iron and display activity against JMJD2A in cell-based studies.

## Results

### Quantitative High-Throughput Screen and Hit Prioritization

**Figure 1:**
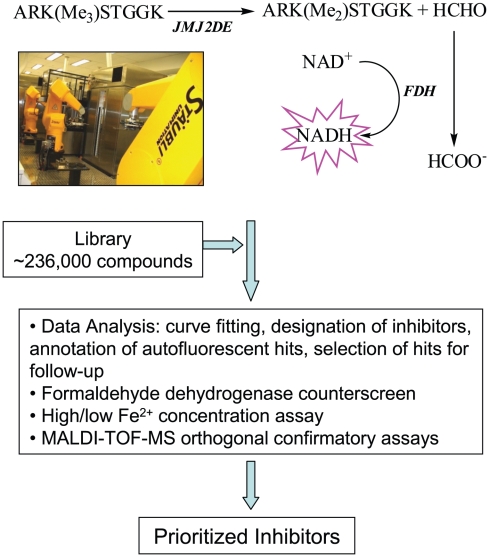
Screening and Hit Prioritization Strategy.

We have reported a miniaturized fluorescence-based assay [20] for high-throughput screening of histone demethylase inhibitors [18]; this assay detects formaldehyde released from the demethylase reaction by converting it to formic acid using formaldehyde dehydrogenase [21]. The oxidation of formaldehyde is coupled to the reduction of NAD^+^ to NADH, which is monitored by fluorescence spectroscopy. In order to identify histone demethylase inhibitor templates, we applied this assay for high-throughput screening of a diverse compound library, comprising the Molecular Libraries Small Molecule Repository (MLSMR) and additional collections, against the 2OG-dependent histone demethylase JMJD2E (KDM4E). JMJD2E was chosen as a representative member of the JMJD2 histone demethylase subfamily with kinetic properties suitable for *in vitro* high-throughput screening. Approximately 236,000 compounds were evaluated in a concentration-response screen using a robotic system. The results of the screen of 1,316 plates were statistically robust, with an average Z′ factor of 0.85 (**Figure S1**), and the screen was completed within 5 days. The screen yielded hits with a wide range of potencies (IC_50_) and with substantial variation in the quality of the corresponding concentration-response curves (efficacy and number of asymptotes), which included samples associated with shallow curves or single-point extrapolated concentration responses; these were assigned as low-confidence actives. Furthermore, the screening results were expected to be affected by interferences arising from factors including compound autofluorescence, noisy/partial concentration responses, false positives acting on the coupled-enzyme component of the assay, and promiscuous inhibitors or potent metal chelators. To this end, initial screening hits were filtered by a combination of cheminformatics (to exclude low-confidence inhibitors and autofluorescent false positives), counterscreening against the coupled-assay component formaldehyde dehydrogenase (FDH), and orthogonal direct detection of inhibition of demethylation by mass spectrometry (**Figure 1**).

Only samples which displayed greater than 40% inhibition at the highest concentration tested were considered as active hits, thus eliminating compounds associated with noisy data and weak partial curves. The initial fluorescence reading for each sample was used to identify compounds contributing high background fluorescence. An arbitrary initial fluorescence reading of 50 relative fluorescence units (RFU) was selected in order to balance the threshold for statistical significance and the need to flag artifacts as rigorously as possible; this represents the average assay signal plus three standard deviations. This was used as a cutoff for exclusion of autofluorescent hits. Application of this step eliminated 21,674 of the actives, a large but not unexpected number due to the blue spectral region of the detection signal [22]. Similarity clustering of the remaining 3,597 actives led to 51 clusters and over 150 singletons. Upon further filtering by potency (cutoff of IC_50_<10 µM and cluster size greater than 4), a total of 157 representative compounds spanning 17 chemical clusters and 83 singletons were prioritized for further studies.

**Figure 2:**
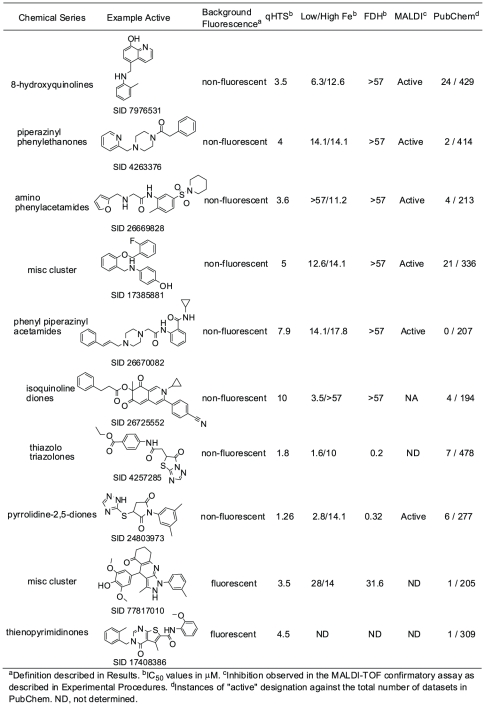
Representative HTS hits. Shown are representative members of clusters along with results from the screen and prioritization experiments.

The selected compounds were then tested to evaluate their effect on the FDH coupling enzyme used for formaldehyde detection. The prioritized compounds were also evaluated in the screening assay using two different ferrous ion concentrations (termed high iron and low iron, respectively), in order to assess their propensity for iron chelation. The results for selected hit series are presented in **Figure 2** (including examples of autofluorescent compounds, such as SID 77817010 and 17408386, some of which otherwise carry attractive chemical features), with the full set of screening results being made freely available in PubChem (PubChem Assay ID 2147). The results for the 157 representative compounds are provided in **Table S1**.

Overall, a small number of prioritized non-fluorescent hits displayed inhibition of the coupling enzyme FDH (e.g. the thiazolo triazolones and pyrrolidine-2,5-dione series, **Figure 2**). This outcome is consistent with a previously-reported screen of the LOPAC^1280^ collection where none of the hits inhibited FDH [18] and with the fact that during the HTS FDH is applied at a concentration approximately 10 times greater than that used in the counterscreen (the counterscreen uses lower FDH concentration in order to maintain reaction linearity and to match the amount of NADH product generated by the two assays). Assaying the prioritized hits against the high- and low-iron conditions (25 and 2.5 µM, respectively) yielded a broad range of IC_50_ shifts, including instances of a decrease in IC_50_ at the higher Fe(II) concentration tested, as well as several increases in IC_50_ of greater than 10-fold at the high Fe(II) concentration. As a reference, the strong iron chelator EDTA was tested and yielded a ratio of 5.9 under these conditions. Furthermore, within series of structurally similar hits, there were considerable variations in the IC_50_ shifts for low/high Fe(II) concentrations. Given these variations and factoring in the relatively narrow range of boundary iron concentrations used in this profile (the latter largely being determined by the need to maintain adequate assay signal), an overreliance on this filtering step to exclude series from further consideration carries the risk of losing viable scaffolds. Thus, we did not exclude hits based on the iron-dependency profile but noted the ratios as a possible indicator for the compounds' mechanism of action (for example, the isoquinoline diones series, **Figure 2** and **Table S1**).

Of the selected 157 hits, 29 representative members were subjected to an orthogonal MALDI-TOF assay to confirm that they were genuine demethylase inhibitors and not inhibitors of FDH. Mass spectrometric assays, which employed micromolar concentrations of JMJD2E and measured direct depletion of a trimethylated lysine-substrate (ARKme3STGGK-NH_2_), confirmed most hit series as JMJD2E inhibitors (**Table S1**). Because of the need to maintain an adequate signal, the MALDI-TOF assay utilized a JMJD2E concentration 20-fold higher than that used in the HTS (2µM); note that this results in decreases in apparent potency as judged by IC_50_ values and the inability to perform curve fitting on some of the responses. Nonetheless, most of the prioritized hit series displayed inhibition in this assay, confirming them as *bona fide* demethylase inhibitors (**Figure 2**). To evaluate the potential for promiscuity of inhibition, the hits presented in **Figure 2** were queried against the PubChem database by noting the instances of an “active” designation versus the total number of datasets in which a particular compound is included. The hits were found to be largely inactive in the 200–400 screens in which they were run, thereby ruling them out as promiscuous inhibitors. In comparison, the flavonoid quercetin, which was previously identified as a JMJD2E inhibitor [18], is reported as being active in 85 assays, inconclusive in 504 assays and inactive in 202 assays.

Among the inhibitor series identified in the qHTS against JMJD2E (**Figure 2**), we selected the 8-hydroxyquinolines (8HQ) for further investigation, with the aims of understanding basic structure-activity relationships (SAR), mode of action, and possible cellular effects. We selected the 8HQ ring system because it is amenable to chemical modification, possesses high ligand efficiency and, because some 8HQ-derivatives have recently been identified as inhibitors of the 2OG dependent hypoxia inducible factor prolyl hydroxylase PHD2 (EGLN1), where both *in vitro* and cell-based potency were demonstrated [23], [24].

**Figure 3:**
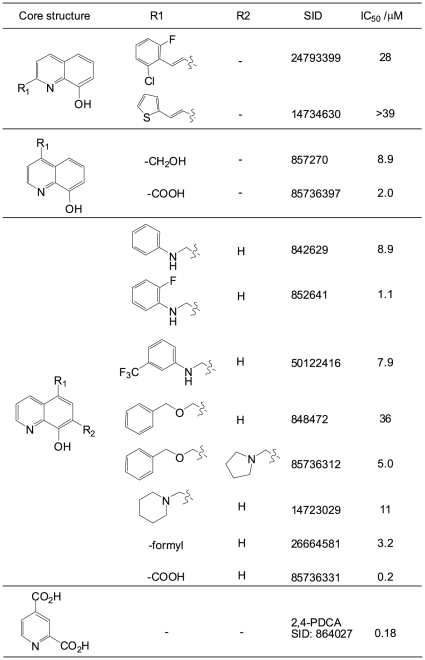
Structure-activity relationships for substituted 8-hydroxyquinolines identified in the qHTS against JMJD2E (qHTS IC_50_ values derived from the FDH-coupled assay). Some substitution patterns were chosen for further medicinal chemistry; IC_50_s for these synthesized compounds are indicated in italics.

A variety of 8HQ substitution patterns were identified from the qHTS. Among these, substitution at the 2-positions showed weaker inhibitory potency, while substitution at the 4-, 5- and 7- positions on the 8HQ ring appeared to result in greater potency (**Figure 3**). The largest number of active analogues were identified within the 5-substituted series, and here a variety of lipophilic and basic substituents were tolerated, in addition to the formyl group. Importantly, note that the trends observed for JMJD2E inhibition are significantly different from those observed in the recent study by Smirnova *et al.* of prolyl hydroxylase inhibitors against the PHD isoforms; in that study, 8HQs with branched substituents at the 7-position were among the best hits [24]. Comparison between the 7-substituted branched 8HQ described in Smirnova *et al.* and the present qHTS dataset reveals one direct structural match, SID 14737227: this compound displayed a shallow response and relatively weak potency against JMJD2E (IC_50_ of 39 µM, **Table S1**).

To investigate the binding mode of the 8HQs to JMJD2E and for comparison with the 2,4-PDCA chemotype, the 4- and 5-carboxy-8HQ analogues were prepared and their identity and purity verified by standard analytical liquid chromatography coupled with mass spectrometry (LC-MS) methods (see Materials and Methods and **Text S1** for details). By analogy with 2,4-PDCA one might expect 4-carboxy-8HQ to bind similarly; however, 5-carboxy-8HQ (SID 85736331) had greater potency with an IC_50_ of 200 nM compared to 2 µM for the 4-carboxy isomer SID 85736397 using the FDH coupled assay (**Figure 3**). Preliminary kinetic studies suggested possible mixed-mode of inhibition for the 5-carboxy-8-HQ with respect to 2-OG (**Figure S2**). In part this might be due to competition not only with 2OG but also with peptide substrate; however, tight-binding competitive inhibitors also display mixed-mode inhibition kinetics when the enzyme concentration used for screening is very close to the apparent K_i_. Thus the IC_50_ for 5-carboxy-8-HQ was measured at a number of different enzyme concentrations; the resulting shift in IC_50_ with increasing enzyme concentration (**Figure S2**) suggests that this compound does display tight-binding inhibitory behaviour. This may account at least in part for the apparent mixed-mode inhibition with respect to 2OG.

### Crystallography

**Figure 4:**
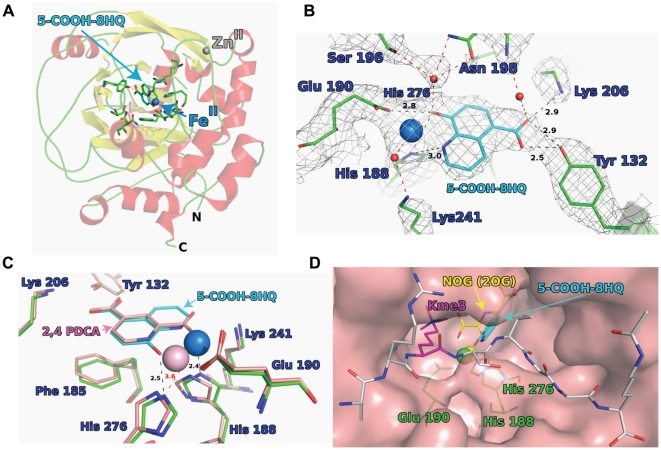
JMJD2A crystal structure complexed with 5-carboxy-8-HQ (SID 85736331). (**A**) active site residues are in green sticks, 5-carboxy-8-hydroxyquinoline as cyan sticks, secondary structure helices in red and sheets in yellow. The Ni(II) ion (replacing Fe(II)), is a blue sphere and the Zn(II) is a grey sphere. (**B**) Binding mode of 5-carboxy-8-hydroxyquinoline in JMJD2A. The experimental 2Fo-Fc electron density is shown as grey mesh (contoured at 1σ). Hydrogen bonds to residues are shown in black. Hydrogen bonds involving waters (red spheres) are shown in red, distances in Å. (**C**) Overlay of JMJD2A crystal structure with the 5-carboxy-8-hydroxyquinoline (residues green, compound cyan, Ni(II) in blue) with the crystal structure with 2,4 PDCA (residues compound and Ni(II) ion are pink, PDB ID: 2VD7). The distances shown are for the 8-hydroxyquinoline structure between: 5-carboxy-8-hydroxyquinoline and His 276, Ni^2+^ and His 188 (in black), His 276 and Ni^2+^ (in red). Distances in Å. (**D**) Surface view of superimposition of 8-hydroxyquinoline 5-acid bound JMJD2A structure with that of JMJD2A bound to histone 3 lysine 9 trimethylated (H3K9me3) substrate peptide (grey and magenta sticks) and the 2-oxoglutarate (2OG) mimetic *N*-oxalylglycine (NOG, yellow sticks), PDB ID: 2OQ6. Binding of the 8-hydroxyquinoline 5-acid (cyan sticks) is likely to prevent binding of 2OG but not directly that of the H3K9me3 residue (in magenta) or other residues in the substrate peptide (in grey). Peptide sequence ARK(me3)STGGK(Ac). Ni^2+^ of structure 2OQ6, yellow sphere; Ni^2+^ of 8-hydroxyquinoline structure, green sphere. The triad of metal coordinating residues are in green sticks. Crystallographic data and refinement parameters are described in **Table S2**.

We were able to crystallize 5-carboxy-8HQ in complex withJMJD2A to investigate the inhibitor interactions within the active site environment. The structure revealed that 5-carboxy-8HQ is positioned in a similar location to the 2OG analogue *N*-oxalylglycine in complex with JMJD2A and Ni(II) (which substitutes for Fe(II)); the 5-carboxy group of 5-carboxy-8HQ and the C-5 carboxylates are positioned to interact with side chains Lys 206, Tyr 132. 5-Carboxy-8HQ is also positioned to coordinate with the active site Ni(II), in a bidentate fashion via its quinoline-nitrogen and 8-hydroxy group (**Figure 4A,B**). Comparison of the 5-carboxy-8HQ structure with that of JMJD2A in complex with the H3K9me3 substrate peptide and *N*-oxalylglycine (PDB ID: 2OQ6) shows that the binding of the 5-carboxy-8HQ is likely to compete with binding of 2OG and, at least, not directly with the peptide substrate (**Figure 4D**) [13], [14], [25]. However, binding of the inhibitor may also indirectly affect substrate binding; this notion is supported in part by the observation of mixed mode inhibition kinetics when 5-carboxy-8HQ was tested with varying concentrations of 2OG (**Figure S2**). Studies in solution on other 2OG oxygenases have shown that binding of inhibitors (and 2OG) can affect the fold away from the active site [26], [27].

An interesting difference between the 5-carboxy-8HQ and 2OG structures, as compared to structures of JMJD2A with peptide or other ligands bound (PDB IDs 2WWJ, 2VD7, 2OQ6, 2OQ7), is that the interaction between the hydroxyl moiety of 5-carboxy-8HQ and His 276 (distance 2.5 Å) appears to replace the ligation bond between the Ni(II) and His 276, shifting the position of the Ni(II) ion away from His 276 (by approximately 1.5 Å).

### Cellular Demethylase Assays

**Figure 5:**
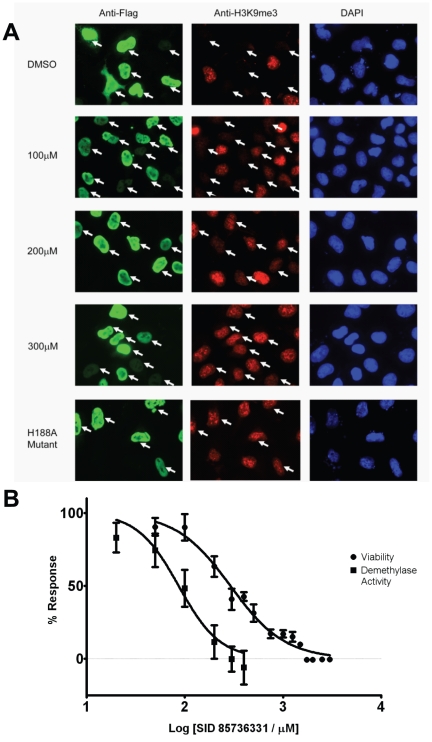
5-Carboxy-8-HQ (SID 85736331) increases H3K9me3 levels in HeLa cells through inhibition of JMJD2A. (**A**) Indirect immunofluorescence with anti-Flag (green), anti-H3K9me3 (red), and DAPI staining (blue) in HeLa cells overexpressing Flag-tagged JMJD2A. DMSO solvent treatment has no effect on JMJD2A demethylase activity (white arrows) while increasing concentrations of 5-carboxy-8-hydroxyquinoline treatment (100 µM to 300µM range shown) resulted in gradual increases in H3K9Me3 levels. The JMJD2A H188A enzymatic mutant does not affect H3K9Me3 levels when overexpressed. (**B**) Quantitation of H3K9me3 levels is shown as squares (▪). Standard deviations are derived from biological triplicates at inhibitor concentrations of 20 µM, 50 µM, 100 µM, 200 µM, 300 µM, and 400 µM treatments. Cytotoxicity (•) was assayed at 50 µM, 100 µM, 200 µM, 300 µM, 400 µM, 500 µM, 750 µM, 1 mM, 1,25 mM, 1.5 mM, 1.75 mM, 2 mM, 2.5 mM, and 3 mM.

To examine if 5-carboxy-8HQ could also inhibit JMJD2 histone demethylase activity in cells we needed to develop an assay to directly measure demethylase activity in vivo. It was previously demonstrated that overexpression of the JMJD2A histone demethylase in cultured cells lead to near complete depletion of cellular H3K9me3 as assessed by indirect immunofluorescence [28]. Based on these observations, we have designed an in cell assay to specifically and quantitatively measure inhibition of JMJD2A histone demethylase activity. To achieve this, a Flag-tagged version of JMJD2A was transiently overexpressed in HeLa cells either in the presence of a vehicle control (DMSO), 1 mM dimethyl-2,4-PDCA (a cell-permeable derivative of 2,4-PDCA), 2.5 mM dimethyloxalylglycine (DMOG, a cell-permeable derivative of *N*-oxalylglycine), or varying concentrations of 5-carboxy-8-HQ. After 24 hours of incubation time cells were fixed and then analyzed by indirect immunofluoresence with Flag antibody to identify the cells overexpressing the demethylase and an antibody recognizing endogenous H3K9me3 to quantify the level of this histone modification. In order to reliably measure the levels of demethylase activity in transfected cells, significantly large cell numbers were required making manual single cell analysis cumbersome and impractical. Therefore, in order to automate this process a series of images were collected for each treatment on a standard epifluorescence microscope (**Figure 5A**) and then submitted to CellProfiler Software for analysis [29]. CellProfiler was configured to analyze the images for DAPI signal, a DNA stain, enabling the program to identify the location of individual cells and create a boundary that delineates the volume of the nuclear compartment. As not all cells in a given field are transfected, the Flag-JMJD2A-expressing cells were identified by quantifying the immunofluoresence signal resulting from the Flag antibody staining and using the mock transfected cells as a baseline for the signal intensity of non-transfected cells. Once the transfected cells were identified, the nuclear H3K9me3 immunofluorescence signal for each cell was quantified by CellProfiler. The levels of H3K9me3 staining intensity were analyzed in the DMSO vehicle treated or inhibitor treated samples. As a control and a means of determining maximal possible inhibition of demethylase activity, cells expressing the JMJD2A H188A catalytically deficient mutant were also quantified in each experiment. The level of demethylase activity inhibition by 5-carboxy-8HQ treatment (**Figure 5B**, squares) was determined by quantifying the immunofluoresence signal from the DMSO treated sample (100% demethylase activity) compared to the maximal theoretical inhibition signal intensity as determined by the H3K9me3 signal in cells expressing the catalytically deficient JMJD2A H188A mutant (0% demethylase activity). For each treatment a minimum of 400 transfected cells was analyzed and the final values of inhibition were derived from inhibition experiments carried out on three separate days (biological triplicates).

Treatment with increasing 5-carboxy-8HQ (SID 85736331) concentrations showed a dose-dependent increase in H3K9me3 fluorescence intensity, demonstrating that H3K9me3 demethylation by JMJD2A is inhibited by 5-carboxy-8HQ in cells (**Figure 5A** **,** **4B** **▪**). The cellular IC_50_ value for 5-carboxy-8-HQ was determined to be 86.5 µM with standard error of 1.16 µM and 95% confidence interval from 63.74 µM to 117.3 µM. The 200 µM 5-carboxy-8HQ treatment inhibition of JMJD2A was greater than treatment with 1 mM PDCA dimethyl ester or 2.5 mM DMOG indicating that 5-carboxy-8HQ is the most potent cellular JMJD2A inhibitor identified to date (**Figure S3**). Notably, this compound does not require modification to a “pro-drug” ester form in order to be active in cells, as do the previously reported *N*-oxalylglycine derivatives [30]. Cytotoxicity testing of the 5-carboxy-8HQ on HeLa cells was conducted with the resazurin/resorufin system for cellular metabolism. Cytotoxicity IC_50_ was determined to be 291.6 µM (**Figure 5B**, •) which is three times the JMJD2A inhibition IC_50_. At 291.6 µM, JMJD2A demethylase activity is considered fully inhibited as H3K9me3 levels are comparable to the H188A catalytic JMJD2A mutant **(** **Figure 5B** (▪) and **Figure S3**). Therefore, the 5-carboxy-8HQ compound functions in a cell to inhibit histone demethylase activity.

### Screening against other 2OG oxygenases

**Figure 6:**
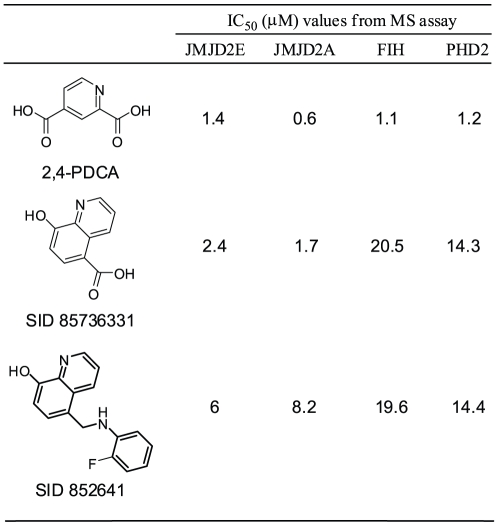
Inhibitor screening against JMJD2 and other human 2OG oxygenases. IC_50_ values from MALDI-TOF MS assay. See **Figure S4** for representative concentration-response curves and MALDI-TOF mass spectra.

To investigate the effect of substitution on the 8HQ template on inhibition of individual 2OG oxygenases, selected 8HQ derivatives were also screened against other human 2OG oxygenases which have been reported to bind 8HQs. Prolyl Hydroxylase Domain 2 (PHD2) and Factor Inhibiting Hypoxia Inducible Factor (FIH) are prolyl and asparaginyl hydroxylases, respectively, that regulate the hypoxic response (for review, see [31]). The compounds in **Figure 6** (representative concentration-response curves and MALDI-TOF spectra shown in **Figure S4**) were screened against JMJD2A, JMJD2E, PHD2 (catalytic domain) and FIH using a MALDI-TOF-MS-based peptide hydroxylation/demethylation assay described previously [14]. 2,4-PDCA, a well-characterized 2OG analogue and inhibitor of many 2OG oxygenases [32], [33], [34], [35], inhibits all the enzymes tested with IC_50_ values in the low micromolar range. Notably, although 5-carboxy-8HQ had a similar potency to 2,4-PDCA against JMJD2E and JMJD2A, it was less active than 2,4-PDCA against FIH and PHD2, suggesting that 5-substituted 8HQ may be a relatively preferred template for, at least, the JMJD2 family of histone demethylases. Testing of this compound and its future analogues against other subfamilies of 2OG-dependent histone demethylases will be of interest; we envisage that this will be part of a new study (production of these enzymes in recombinant form in *E. coli* has not been reported).

## Discussion

We investigated whether a screen of a large-size diverse small molecule collection could yield novel scaffolds directed at the histone demethylase family that would be useful in *in vitro* studies. Following the initial screen of nearly a quarter of a million compounds, we carried out a comprehensive filtering strategy in order to derive high-confidence inhibitor chemotypes for this family of epigenetic regulators. The results from this screening and hit triaging study have been placed in the public domain (PubChem), with the aim of providing the research community with a wide range of leads for the development of improved agents.

In the present study, we selected one series, the 8-hydroxyquinolines, for further investigations, including preliminary SAR investigations which yielded an analogue with improved potency. X-ray crystallography was used to rationalize the mode of action of this novel demethylase-directed scaffold: 5-carboxy-8HQ occupies the 2OG binding site, and kinetic data indicate that it may indirectly affect the binding of the peptide substrate. A comparison between the present structure and structures of JMJD2A with substrates or other ligands, such as 2,4-PDCA, highlights a notable difference in the ligation of the active-site metal and the His276 residue: the hydroxyl group from the inhibitor interacts with His276 while the nickel ion (substitution for iron) formerly bound to the same His is now shifted away from that amino acid. Although it is unclear whether the observed shift in metal position occurs in solution with Fe(II) complexed at the active site, it highlights the possibility that inhibition of 2OG oxygenases by metal-chelation at their active sites may involve movement of the metal.

Our findings, and those of others [23], [36], demonstrate that 8HQ may provide an attractive generic template for generating inhibitors of 2OG oxygenases, and that inhibitory selectivity can be achieved through substitutions to the 8HQ core. This proposal is supported by the distinct patterns of 8HQ substitution determined to be important for JmjC-domain demethylase activity here (**Figure 3**) and the recent study of Smirnova *et al.* where a branched substituent at the 7-position was found to be important for 8HQ activity against the related PHDs. It is anticipated that further medicinal chemistry efforts supported by structure-based design will thus enable increases in inhibitor potency and selectivity amongst 2OG oxygenases, and potentially among the different histone demethylase subfamilies.

Finally, having verified the 8HQ mode of interaction with the demethylase targets *in vitro*, we evaluated the effect of 5-carboxy-8HQ on histone demethylase activity inside cells. Unlike the cases of histone methyltransferases or histone deacetylases where multiple examples of loci under direct control of the corresponding HMTs or HDACs exist, there is no good target gene-based system for histone demethylase studies. We therefore developed a transfection-based assay to test the inhibitor's effect within the context of a selectively-upregulated demethylase. Changes in K9me3 levels can only be mediated by inhibition of JMJD2 demethylases (no other demethylases identified to date are capable of demethylating K9me3) and because the assay quantitates the degree of histone trimethylation at the H3K9 locus only within cells that overexpress demethylase, this measurement is relatively insensitive to the potential effects of the small molecule on the level of transcription of the underlying gene or on other targets within the cell. In the assay, the present 5-carboxy-8HQ inhibitor displayed a strong concentration-dependent effect, restoring the trimethylation pattern at the H3K9 locus in cells overexpressing the JMJD2A/KDM4A demethylase. Prior to this work, inhibition of demethylase activity had only been shown using weak nonselective agents, such as DMOG (pro-drug form), at millimolar doses. The 40-fold stronger potency of the present lead, combined with its improved selectivity profile *in vitro* and the availability of a range of analogues, are expected to make this compound a good starting point for the development of a potent and selective agent to study epigenetic regulation in cellular context.

## Materials and Methods

### Reagents

Ferrous ammonium sulfate (FAS), (+)-sodium *L*-ascorbate (SA), β-nicotinamide adenine dinucleotide hydrate (NAD^+^), Tween-20, formaldehyde dehydrogenase from *Pseudomonas putida* (FDH), and disodium 2OG were from Sigma-Aldrich (St. Louis, MO). Dimethylsulfoxide certified ACS grade (DMSO) was from Fisher, Inc. HEPES buffer was obtained from Gibco. The trimethylated histone peptide substrate ARK(me_3_)STGGK was synthesized and HPLC-purified either in-house using a CS-Bio CS336S automated peptide synthesizer or by the Tufts University Core Facility (Boston, MA). Synthetic methods for the preparation of 4-carboxy-8-hydroxyquinoline and 5-carboxy-8-hydroxyquinoline are provided in **Text S1**. Black solid-bottom 1,536-well assay plates were from Greiner Bio-One (Monroe, NC).

### Enzymes

The catalytic domain of human JMJD2E (residues 1–337) was produced as an *N*-terminally His_6_-tagged protein in *E. coli*, and purified by Ni-affinity chromatography and size-exclusion chromatography, and stored at a concentration of 60 mg/mL in HEPES 50 mM NaCl 500 mM pH 7.5, as reported [13]. JMJD2A was expressed and purified as reported [25]. Factor Inhibiting Hypoxia Inducible Factor (FIH) was prepared by Ni affinity followed by gel filtration chromatography [37] and Prolyl Hydroxylase Domain 2 (PHD2)^181–486^ was prepared by cation exchange chromatography followed by gel filtration chromatography as described [38].

### Compound library and quantitative high-throughput screen (qHTS)

A library of 236,376 structurally diverse compounds was tested for JMJD2E inhibition. Compounds were serially diluted 1∶5 in DMSO to yield 7 concentrations (0.64 µM–10 mM) and were formatted in 1536-well plates. The library comprised the Molecular Libraries Small Molecule Repository (MLSMR, http://mlsmr.glpg.com/MLSMR_HomePage/), as well as several sets of bioactive molecules, approved drugs, and various diverse collections. Details on the composition of the screening library and on the preparation of the library for qHTS are provided elsewhere [39]. JMJD2E-catalyzed (100 nM) demethylation of a trimethylated histone H3 peptide was measured in the presence of the potential inhibitor (3.7 nM–57 µM) as reported [18] in a fluorescence-based enzymatic assay using the coupling enzyme formaldehyde dehydrogenase (FDH) [20]. Compounds were added by pintool transfer of 23 nL stock in DMSO to 3 µL of enzyme in 50 mM HEPES buffer, pH 7.5, containing 0.01% Tween-20 dispensed in black solid-bottom 1536-well plates (final 0.67% DMSO) and pre-incubated for 15 min. Reactions were initiated with 1 µL addition of peptide substrate solution containing final concentrations of 10 µM FAS, 50 µM 2OG, 0.25 U/mL FDH, 0.25 mM NAD^+^, and 50 µM peptide substrate (ARK(me3)STGGK). A ViewLux imager (PerkinElmer, Waltham, MA) was used to measure NADH fluorescence (λ_ex_ 340 nm, λ_em_ 450 nm) immediately after substrate addition and following 30 min incubation at room temperature. To record any systematic drifts in assay behavior (signal window and noise), assay plates containing no library compounds but only DMSO as a vehicle control were tested periodically (every ∼60 plates) throughout the screen. The screen was conducted on a fully-integrated robotic system (Kalypsys, Inc., San Diego, CA) as described in detail elsewhere [40].

Data were normalized to the basal response (0% activity) in the absence of enzyme and the maximal response (100% activity) in the absence of compound. Using the Hill equation, dose-response curves and IC_50_ values were automatically generated for each compound tested with software developed internally (www.ncgc.nih.gov/pub/openhts/curvefit/). Clustering of active compounds by structural similarity was performed using Leadscope Hosted Client (Leadscope Inc., Columbus, OH). The initial well fluorescence (first kinetic read) associated with each sample was stored in a database and used in the cheminformatics analysis to identify autofluorescent compounds interfering with the assay signal.

### Hit Confirmation

To minimize the possibility of false positives arising through inhibition of the FDH coupling enzyme, active compounds were tested in a reaction containing 0.025 U/mL FDH enzyme and the substrates 0.25 mM NAD^+^ and 10 µM formaldehyde, using a fluidic protocol identical to that used for qHTS. Compounds were also tested for their potential to act as iron chelators in solution by measuring inhibition of JMJD2E activity under both high and low iron conditions (25 and 2.5 µM Fe(II), respectively). The ratio of IC_50_ values under high iron to low iron was calculated for each compound. Compounds that exhibited ratios >3.0 were identified as potential chelators, where the ratio of 3.0 was based arbitrarily on the ratio of 5.9 obtained for the strong iron chelator EDTA.

### Matrix-Assisted Laser Desorption/Ionization-Time-of-flight (MALDI-TOF) Mass Spectrometry

JMJD2 assays were carried out as reported [13]. In brief, an assay reaction consisted of JMJD2 (1 µM (Figure 5) or 2 µM in qHTS hit confirmation (**Table S1**)), FAS (10 µM), SA (100 µM), 2OG (10 µM) ARK(me_3_)STGGK peptide (10 µM), in 50 mM HEPES (pH 7.5) with varying concentrations of inhibitors (final DMSO concentration was 5%). Initially, JMJD2, Fe(II), ascorbate and inhibitors were pre-incubated for 15 min prior to the addition of peptide and 2OG. The reaction was incubated for 30 min before 1∶1 quenching with methanol followed by addition of four volumes of 20 mM triammonium citrate. PHD2 and FIH assays were carried out as described [41], [42] with minor modifications. PHD2 assay consisted of PHD2 (1 µM), FAS (10 µM), SA (100 µM), 2OG (60 µM) and HIF-1α^556–574^CODD peptide (50 µM), in 50 mM HEPES (pH 7.5) with varying concentrations of inhibitors (final DMSO concentration was 5%). Initially, PHD2, Fe(II), ascorbate and inhibitors were pre-incubated for 15 min prior to the addition of peptide and 2OG. The reaction was incubated for 30 min before 1∶1 quenching with 1% TFA followed by addition of three volumes of 20 mM triammonium citrate. FIH assay consisted of FIH (1 µM), FAS (10 µM), SA (100 µM), 2OG (100 µM) and HIF-1α^786–826^CAD peptide (100 µM), in 50 mM HEPES (pH 7.5) with varying concentrations of inhibitors (final DMSO concentration was 5%). Initially, FIH, Fe(II), ascorbate, and inhibitors were pre-incubated for 15 min prior to the addition of peptide and 2OG. The reaction was incubated for 30 min before 1∶1 quenching with 200 mM HCl followed by addition of two volumes of dH_2_O. The diluted assay mixture (1 µL) from JMJD2 and PHD2 assays were mixed with 1 µL of α-cyano-4-hydroxycinnamic acid and 1 µL of FIH assay mixture was mixed with 1 µL of sinapinic acid matrix, and assessed by matrix-assisted laser desorption/ionization-time of flight (MALDI-TOF).

### Crystallography

JMJD2A was diluted with gel filtration buffer (10 mM HEPES pH 7.5, 500 mM NaCl and 5% glycerol) to a concentration of 10 µM (0.5 mg/mL). The 5-carboxy-8-HQ was added to the protein solution to a final concentration of 100 µM (0.1% DMSO). The solution was then concentrated in a 4 ml centrifugal concentrator (Centricon™, Millipore, MWC 10 kDa) to a final protein concentration of 250 µM (11 mg/mL). This solution was then used for crystallization trials. Crystals were grown by vapor diffusion at 4°C in 300 nL sitting drops with a 1∶2 ratio of protein to well solution (100 mM Na citrate pH 5.5, 4 mM NiCl_2_ and 20% w/v PEG 3350). Crystals were cryoprotected by transferring to a solution of mother liquor supplemented with 2 mM 5-carboxy-8-hydroxyquinoline and 25% v/v glycerol before being flash frozen in liquid N_2_. Data were collected from a single crystal at 100 K at the Diamond Light Source, beamline I04 using a beam with wavelength 0.97630 Å. The data were processed with iMOSFLM [43] and SCALA [44] followed by molecular replacement using Phaser [45]. Refinement was carried out with PHENIX (version 1.6) [46] with iterative rebuilding of the model using Coot [47]. The Final R and R_free_ values were 18.0% and 22.6% respectively. All residues were in acceptable regions of a Ramachandran plot as calculated by MolProbity [48]. Crystallographic statistics are shown in **Table S2**.

### Cellular Demethylase Assay

HeLa cells were maintained in DMEM media supplemented with 10% FBS and penicillin/streptomycin. Cells were transiently transfected with either Flag-tagged JMJD2A or the H188A catalytic mutant of JMJD2A using Fugene HD. Inhibition studies were initiated 4 hr after cellular transfection and compounds were added to a final concentration of 20–400 µM in 0.5% DMSO. H3K9me3 levels were measured using immunofluorescence staining as described [28] in cells following 24 hr incubation with compound. All cells were stained with an anti-Flag mouse monoclonal antibody (M2; Sigma F1804), rabbit anti-H3K9me3 (Abcam Ab8898), and DAPI for DNA. FITC conjugated mouse and Rhodamine conjugated rabbit secondary antibodies were used to fluorescently label the Flag and H3K9me3 primary antibodies. Image analysis was conducted using CellProfiler [29]. Transfected cells were identified as those cells with higher Flag immunofluorescence than mock transfected cells. Data were normalized by setting DMSO treated JMJD2A transfected cells to 100% demethylase activity and the catalytic mutant H188A transfected cells to 0% activity. The data shown are averages of 3 or more biological replicates per concentration. Cytotoxicity testing was done with CellTiter-Blue (Promega) according to the manufacturer's directions.

## Supporting Information

Figure S1 **HTS statistical performance.** A robust Z′ factor was maintained throughout the HTS for JMJD2E inhibitors.
(PDF)

Figure S2 **Kinetics and Mode of Inhibition of 5-carboxy-8-8HQ against JMJD2E.** FDH assay was carried out at 2µM JMJD2E, 200 µM ARK(me3)STGGK peptide (excess of Km) and varying 2OG concentrations.
(PDF)

Figure S3 **Cell-based demethylation assay.** **(A)** Quantitation of H3K9me3 levels. 5-Carboxy-8-hydroxyquinoline (SID 85736331) treatments were at 20µM, 50µM, 100µM, 200µM, 300µM, and 400µM compared to 2.5mM Dimethyloxalylglycine (DMOG) and 1mM dimethyl-2,4-PDCA. Treatment with 200µM 5-carboxy-8HQ inhibited JMJD2A demethylase activity to approximately 10% which is greater than treatment with either 2.5mM DMOG (approximately 20% activity) or 1mM dimethyl-2,4-PDCA (approximately 35% activity). **(B)** Indirect immunofluorescence with anti-Flag (green), anti-H3K9me3 (red), and DAPI staining (blue) for DNA in HeLa cells overexpressing Flag-tagged JMJD2E or JMJD2E catalytic mutant.
(PDF)

Figure S4 **Inhibitor screening against JMJD2 and other human 2OG oxygenases.** (A) Representative IC_50_ curve for JMJD2A inhibition by SID 85736331. (B) Representative MALDI-TOF mass spectrum for JMJD2A demethylation reactions by MALDI-TOF MS at different inhibitor concentrations.
(PDF)

Table S1 **Inhibitory activities of HTS Hits.** Provided as a separate Excel spreadsheet.
(XLSM)

Table S2 **Crystallographic data and refinement parameters.**
(DOC)

Text S1 **Synthetic and Analytical Procedures.**
(DOC)
