# Supplementary material for: Quantitative High-Throughput Screening Identifies 8-Hydroxyquinolines as Cell-Active Histone Demethylase Inhibitors
Source: PLoS One. 2010 Nov 23;5(11):e15535. doi: 10.1371/journal.pone.0015535 (PMC2990756; doi:10.1371/journal.pone.0015535)

Supplemental Figure S1. HTS statistical performance. A robust Z′ factor was maintained throughout the HTS for JMJD2E inhibitors.

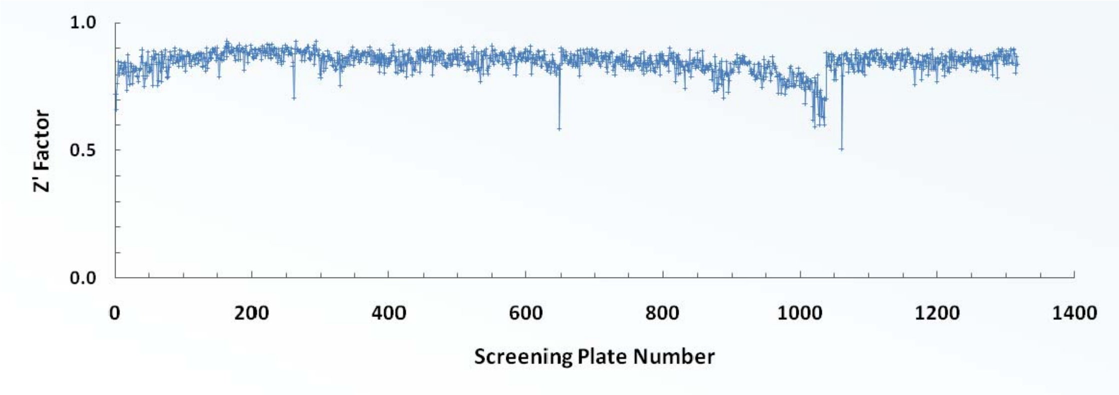

Supplement: Figure S1 — HTS statistical performance. A robust Z′ factor was maintained throughout the HTS for JMJD2E inhibitors. (PDF) [file pone.0015535.s001.pdf]
