# Supplementary material for: Quantitative High-Throughput Screening Identifies 8-Hydroxyquinolines as Cell-Active Histone Demethylase Inhibitors
Source: PLoS One. 2010 Nov 23;5(11):e15535. doi: 10.1371/journal.pone.0015535 (PMC2990756; doi:10.1371/journal.pone.0015535)

Supplemental Figure S2. **Kinetics and Mode of Inhibition of 5-carboxy-8-8HQ against JMJD2E**. FDH assay was carried out at 2µM JMJD2E, 200 µM ARK(me3)STGGK peptide (excess of Km) and varying 2OG concentrations.

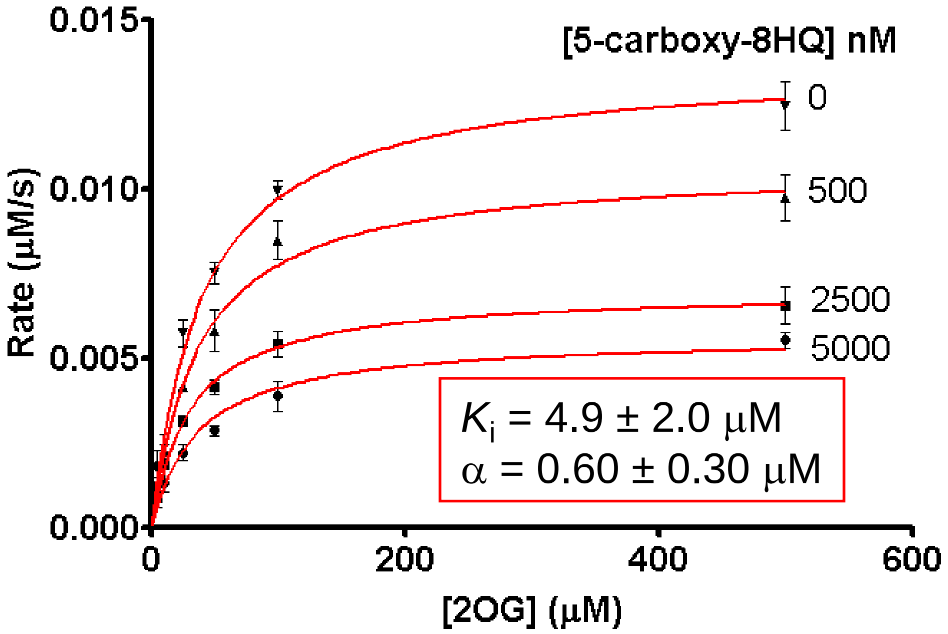

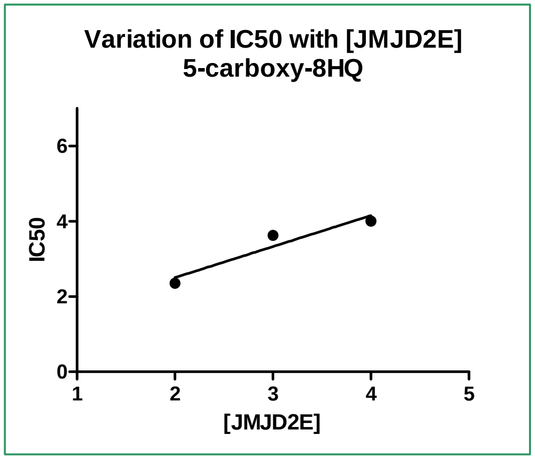

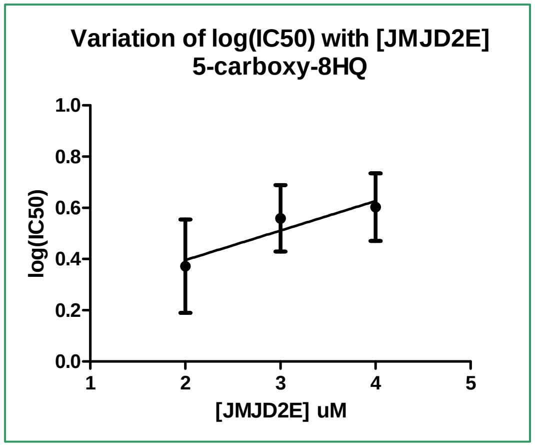

Supplement: Figure S2 — Kinetics and Mode of Inhibition of 5-carboxy-8-8HQ against JMJD2E. FDH assay was carried out at 2µM JMJD2E, 200 µM ARK(me3)STGGK peptide (excess of Km) and varying 2OG concentrations. (PDF) [file pone.0015535.s002.pdf]
