# Supplementary material for: Quantitative High-Throughput Screening Identifies 8-Hydroxyquinolines as Cell-Active Histone Demethylase Inhibitors
Source: PLoS One. 2010 Nov 23;5(11):e15535. doi: 10.1371/journal.pone.0015535 (PMC2990756; doi:10.1371/journal.pone.0015535)

Supplemental Figure S3. Cell-based demethylation assay. (A) Quantitation of H3K9me3 levels. 5-Carboxy-8-hydroxyquinoline (SID 85736331) treatments were at 20µM, 50µM, 100µM, 200µM, 300µM, and 400µM compared to 2.5mM Dimethyloxalylglycine (DMOG) and 1mM dimethyl-2,4-PDCA. Treatment with 200µM 5-carboxy-8HQ inhibited JMJD2A demethylase activity to approximately 10% which is greater than treatment with either 2.5mM DMOG (approximately 20% activity) or 1mM dimethyl-2,4-PDCA (approximately 35% activity). **(B)** Indirect immunofluorescence with anti-Flag (green), anti-H3K9me3 (red), and DAPI staining (blue) for DNA in HeLa cells overexpressing Flag-tagged JMJD2E or JMJD2E catalytic mutant.

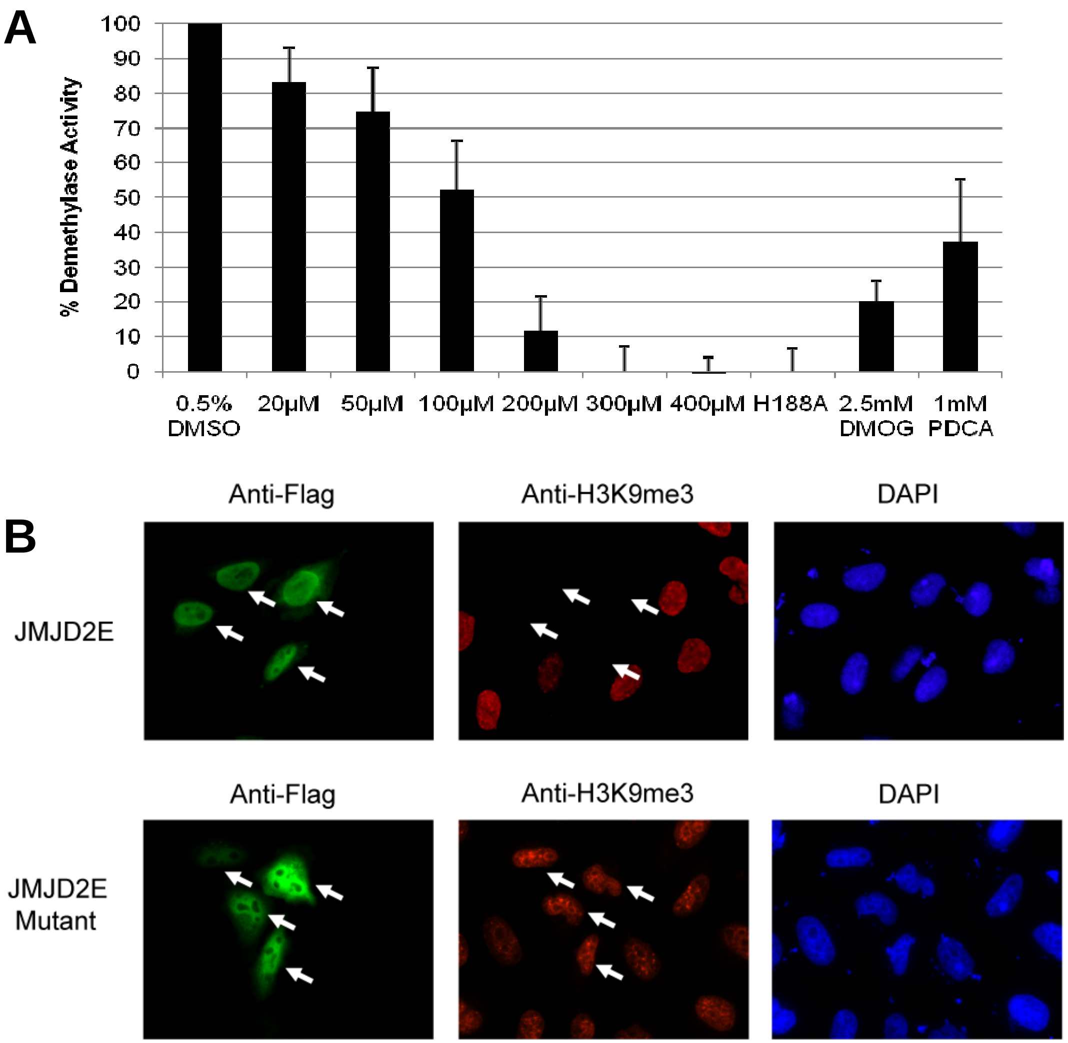

Supplement: Figure S3 — Cell-based demethylation assay. (A) Quantitation of H3K9me3 levels. 5-Carboxy-8-hydroxyquinoline (SID 85736331) treatments were at 20µM, 50µM, 100µM, 200µM, 300µM, and 400µM compared to 2.5mM Dimethyloxalylglycine (DMOG) and 1mM dimethyl-2,4-PDCA. Treatment with 200µM 5-carboxy-8HQ inhibited JMJD2A demethylase activity to approximately 10% which is greater than treatment with either 2.5mM DMOG (approximately 20% activity) or 1mM dimethyl-2,4-PDCA (approximately 35% activity). (B) Indirect immunofluorescence with anti-Flag (green), anti-H3K9me3 (red), and DAPI staining (blue) for DNA in HeLa cells overexpressing Flag-tagged JMJD2E or JMJD2E catalytic mutant. (PDF) [file pone.0015535.s003.pdf]
