# Supplementary material for: Quantitative High-Throughput Screening Identifies 8-Hydroxyquinolines as Cell-Active Histone Demethylase Inhibitors
Source: PLoS One. 2010 Nov 23;5(11):e15535. doi: 10.1371/journal.pone.0015535 (PMC2990756; doi:10.1371/journal.pone.0015535)

Supplemental Figure S4. Inhibitor screening against JMJD2 and other human 2OG oxygenases. (A) Representative IC_{50} curve for JMJD2A inhibition by SID 85736331. (B) Representative MALDI-TOF mass spectrum for JMJD2A demethylation reactions by MALDI-TOF MS at different inhibitor concentrations.

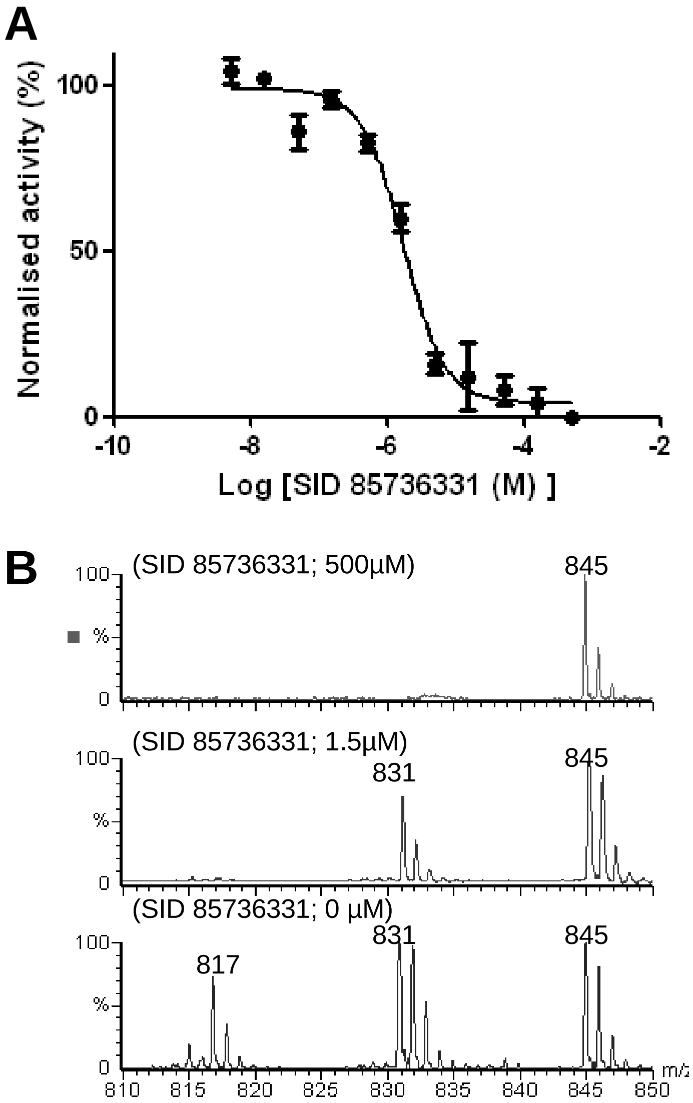

Supplement: Figure S4 — Inhibitor screening against JMJD2 and other human 2OG oxygenases. (A) Representative IC50 curve for JMJD2A inhibition by SID 85736331. (B) Representative MALDI-TOF mass spectrum for JMJD2A demethylation reactions by MALDI-TOF MS at different inhibitor concentrations. (PDF) [file pone.0015535.s004.pdf]
