# Supplementary material for: Quantitative High-Throughput Screening Identifies 8-Hydroxyquinolines as Cell-Active Histone Demethylase Inhibitors
Source: PLoS One. 2010 Nov 23;5(11):e15535. doi: 10.1371/journal.pone.0015535 (PMC2990756; doi:10.1371/journal.pone.0015535)
Supplement: Table S2 — Crystallographic data and refinement parameters. (DOC) [file pone.0015535.s006.doc]

| X-Ray Source | Diamond Light Source Beam I04 | |
| --- | --- | --- |
| PDB ID | 3NJY | |
| Wavelength | 0.97860 Ǻ | |
| Space group | P21212 | |
| Unit Cell Dimensions (a b c) | 100.55 148.99 56.91 | |
| R value (Working and Test Set) | 18.2 % | |
| R value (Working Set) | 18.0 % | |
| Free R Value | 22.6 % | |
| Free R Value Test Set Size | 5.1% | |
| Average Mosaicity | 0.82 | |
|  | **Overall** | **OuterShell** |
| Low resolution limit | 41.24 Ǻ | 2.74 Ǻ |
| High resolution limit | 2.6 Ǻ | 2.6 Ǻ |
| Rmerge | 11.1 % | 40.0 % |
| Rmeas | 12.5% | 45.8% |
| Rpim | 5.7% | 21.9% |
| Total number of observations | 119064 | 16405 |
| Total number unique | 26805 | 3750 |
| Mean((I)/sd(I)) | 9.4 | 4.2 |
| Completeness | 99.0 % | 97.1 % |
| Multiplicity | 4.4 | 4.4 |

**Supplemental Table S2.** Crystallographic data and refinement parameters.
