# Supplementary material for: Quantitative High-Throughput Screening Identifies 8-Hydroxyquinolines as Cell-Active Histone Demethylase Inhibitors
Source: PLoS One. 2010 Nov 23;5(11):e15535. doi: 10.1371/journal.pone.0015535 (PMC2990756; doi:10.1371/journal.pone.0015535)
Supplement: Text S1 — Synthetic and Analytical Procedures. (DOC) [file pone.0015535.s007.doc]

**Supplemental Experimental Procedure**

**General Chemistry.** Unless otherwise stated, all reactions were carried out under an atmosphere of dry argon or nitrogen in dried glassware. Indicated reaction temperatures refer to those of the reaction bath, while room temperature (rt) is noted as 25 ^o^C. All solvents were of anhydrous quality purchased from Aldrich Chemical Co. and used as received. Commercially available starting materials and reagents were purchased from Aldrich and were used as received.

Analytical thin layer chromatography (TLC) was performed with Sigma Aldrich TLC plates (5 x 20 cm, 60 Å, 250 µm). Visualization was accomplished by irradiation under a 254 nm UV lamp. Chromatography on silica gel was performed using forced flow (liquid) of the indicated solvent system on Biotage KP-Sil pre-packed cartridges and using the Biotage SP-1 automated chromatography system. ^1^H- and ^13^C NMR spectra were recorded on a Varian Inova 400 MHz spectrometer. Chemical shifts are reported in ppm with the solvent resonance as the internal standard (CDCl_3_ 7.26 ppm, 77.00 ppm, DMSO-*d*_6_ 2.49 ppm, 39.51 ppm for ^1^H, ^13^C respectively). Data are reported as follows: chemical shift, multiplicity (s = singlet, d = doublet, t = triplet, q = quartet, br = broad, m = multiplet), coupling constants, and number of protons. Low resolution mass spectra (electrospray ionization) were acquired on an Agilent Technologies 6130 quadrupole spectrometer coupled to the HPLC system. High resolution mass spectral data was collected in-house using and Agilent 6210 time-of-flight mass spectrometer, also coupled to an Agilent Technologies 1200 series HPLC system. If needed, products were purified via a Waters semi-preparative HPLC equipped with a Phenomenex Luna® C18 reverse phase (5 micron, 30 x 75 mm) column having a flow rate of 45 mL/min. The mobile phase was a mixture of acetonitrile (0.025% TFA) and H_2_O (0.05% TFA), and the temperature was maintained at 50 ^o^C.

Samples were analyzed for purity on an Agilent 1200 series LC/MS equipped with a Luna® C18 reverse phase (3 micron, 3 x 75 mm) column having a flow rate of 0.8-1.0 mL/min over a 7-minute gradient and a 8.5 minute run time. Purity of final compounds was determined to be >95%, using a 3 µL injection with quantitation by AUC at 220 and 254 nm (Agilent Diode Array Detector).

**8-hydroxyquinoline-4-carboxylic acid (4-COOH-8HQ).** A mixture of 4-bromo-8-hydroxyquinoline[[1]](#endnote-2) (0.6 g, 2.7 mmol, 1.0 equiv), 2-(trimethylsilyl)ethanol (0.57 mL, 4.0 mmol, 1.5 equiv) and triphenylphosphine (1.4 g, 5.4 mmol, 2 equiv) in THF (15 mL) was added DIAD (1.1 mL, 5.4 mmol, 2 equiv) dropwise at 0 ^o^C. The reaction mixture was warmed to room temperature and stirred overnight. The solvent was evaporated and the crude residue was purified on a Biotage® silica gel column. Elution with 25% ethyl acetate in hexanes gave the product. Yield: 92% (0.8 g). 4-bromo-8-(2-(trimethylsilyl)ethoxy)quinoline (0.23 g, 0.71 mmol), 1,3-bis(diphenylphosphino)propane (58 mg, 0.14 mmol, 0.2 equiv) and NEt_3_ (0.4 mL, 2.8 mmol, 4 equiv) were dissolved in a mixture of DMSO (2 mL) and MeOH (2 mL) and degassed with argon for 10 min. Pd(OAc)_2_ (16 mg, 0.07 mmol, 0.1 equiv) was then added and stirred under a CO(g) atmosphere (CO balloon) at 60 ^o^C for 16 h. The crude reaction mixture was extracted with dichloromethane, washed with water, saturated ammonium chloride (aq) and brine. The crude product was purified on a Biotage® silica gel column. Elution with 25% ethyl acetate in hexanes gave the product. Yield: 79% (0.17 g). The pure product (0.17 g, 0.56 mmol, 1 equiv) in THF (5 mL) was deprotected with TBAF (1 M in THF) (2.80 mL, 2.8 mmol, 5 equiv) at RT overnight. Lithium hydroxide and few drops of water were then added and stirred at for 1 h. The crude product was purified in a preparative HPLC to get the pure product. LC-MS: rt (min) = 1.08; ^1^H NMR (DMSO-*d_6_*) δ 7.22 (d, *J* = 7.6 Hz, 1H), 7.61 (t, *J* = 8.0 Hz, 1H), 8.01 (d, *J* = 4.4 Hz, 1H), 8.09 (d, *J* = 8.8 Hz, 1H) and 9.01 (d, *J* = 4.4 Hz, 1H); note: exchangable H’s not observed (phenolic and carboxylic acid); HRMS (ESI) *m*/*z* 189.0429 (M)^+^ (C_10_H_7_NO_3_ requires 189.0426).

**8-hydroxyquinoline-5-carboxylic acid (5-COOH-8HQ).** A mixture of 5-bromo-8-hydroxyquinoline (4.0 g, 17.9 mmol, 1.0 equiv), 2-(trimethylsilyl)ethanol (3.8 mL, 27 mmol, 1.5 equiv) and triphenylphosphine (9.4 g, 35.7 mmol, 2.0 equiv) in a mixture of THF (40 mL) and toluene (40 mL) was added DIAD (7.4 mL, 35.7 mmol, 2.0 equiv) dropwise at 0 ^o^C. The reaction mixture was warmed to room temperature and stirred overnight. The solvent was evaporated and the crude residue was purified on a Biotage® silica gel column. Elution with 25% ethyl acetate in hexanes gave the product. Yield: 88% (5.8 g). 5-bromo-8-(2-(trimethylsilyl)ethoxy)quinoline (5.1 g, 15.7 mmol, 1,3-bis(diphenylphosphino)propane (1.3 g, 3.2 mmol, 0.2 equiv) and NEt_3_ (8.8 mL, 63 mmol, 4 equiv) were dissolved in a mixture of DMSO (40 mL) and MeOH (40 mL) and degassed with argon for 10 minutes. Pd(OAc)_2_ (350 mg, 1.6 mmol, 0.1 equiv) was then added and stirred under saturated CO (g) atmosphere (CO balloon) at 60 ^o^C for 16 h. The crude reaction mixture was extracted with dichloromethane, washed with water, saturated ammonium chloride (aq) and brine. The crude product was purified on a Biotage® silica gel column. Elution with 25% ethyl acetate in hexanes gave the product. Yield: 96% (4.8 g). The pure product (2.0 g, 2 mmol, 1 equiv) was deprotected with TFA (5 mL, 66 mmol, 10 equiv) in dichloromethane (8 mL) by heating in µW at 100 ^o^C for 30 min. The solvent was removed and the crude product was neutralized with aq. sodium bicarbonate solution and extracted with dichloromethane. The organic layer was dried over magnesium sulfate and upon removal of the solvent afforded sufficiently pure product. The ester (0.50 g, 2.5 mmol) and lithium hydroxide (0.30 g, 12.3 mmol, 5 equiv) in a mixture of THF (16 mL), methanol (8 mL) and water (3 mL) was stirred at 60 ^o^C for 10 h. The crude product was purified in a preparative HPLC to get the pure product. LC-MS: rt (min) = 2.35; ^1^H NMR (400 MHz, DMSO-*d*_6_) d 7.12 (d, 1H, *J* = 8.0 Hz), 7.70 (dd, 1H, *J* = 8.8 and 4.0 Hz), 8.25 (d, 1H, *J* = 8.4 Hz), 8.91 (dd, 1H, *J* = 4.0 and 1.6 Hz), and 9.46 (dd, 1H, *J* = 8.8 and 1.6 Hz); note: exchangable H’s not observed (phenolic and carboxylic acid); HRMS (ESI) *m*/*z* 190.0501 (M + H)^+^ (C_10_H_8_NO_3_ requires 190.0499).

1. (a) Irving, H.; Pinnington, A. R. Bromine-substituted derivatives of 8-hydroxyquinoline. *J. Chem. Soc.* **1957,** 290-295. (b) Gershon, H.; Clarke, D. D. Improved syntheses of some monochloro- and monobromo-8-quinolinols. *Monatsh. Chem.* **1991,** *122*, 935-941. [↑](#endnote-ref-2)
